# Correlation of Hematological Indices and Acute-Phase Reactants in Rheumatoid Arthritis Patients on Disease-Modifying Antirheumatic Drugs: A Retrospective Cohort Analysis

**DOI:** 10.3390/jcm12247611

**Published:** 2023-12-11

**Authors:** Yu-Jen Pan, Kuei-Ying Su, Chih-Lung Shen, Yi-Feng Wu

**Affiliations:** 1 Division of Allergy, Immunology and Rheumatology, Hualien Tzu Chi Hospital, Buddhist Tzu Chi Medical Foundation, Hualien 970473, Taiwan; pyj@tzuchi.com.tw (Y.-J.P.); kueiying.su@tzuchi.com.tw (K.-Y.S.); winpower0105@gmail.com (C.-L.S.); 2 School of Medicine, Tzu Chi University, Hualien 970374, Taiwan; 3 Department of Hematology and Oncology, Hualien Tzu Chi Hospital, Buddhist Tzu Chi Medical Foundation, Hualien 970473, Taiwan

**Keywords:** hematological indices, acute-phase reactants, rheumatoid arthritis, treatment, cohort study

## Abstract

Acute-phase markers are often used to evaluate the disease activity of rheumatoid arthritis (RA). Occasionally, the serum levels of acute-phase reactants remain normal in patients with obvious inflamed joints. Hematological indices derived from complete blood counts have been shown to correlate with disease activity. This provides a potential practical implementation in daily practice. Only a few studies have evaluated the relation between hematological indices and novel RA treatment (i.e., biological and targeted synthetic disease-modifying antirheumatic drugs (b/tsDMARDs); no research has examined the changes in hematological indices in RA treatments longitudinally. We conducted a retrospective study involving 273 RA patients with b/tsDMARD treatment and followed them for at least a year. Baseline, 3-month, and 6-month lab data were collected. The results indicated a reduction in the neutrophil–lymphocyte ratio (NLR), platelet–lymphocyte ratio (PLR), monocyte–lymphocyte ratio (MLR), and systemic immune-inflammation index (SII) post-treatment. Higher baseline PLRs and SIIs were associated with a more significant reduction in ESR at three months (η^2^ = 0.03/0.13, *p* = 0.21/0.023). NLR and SII correlated with CRP moderately at three months (r = 0.373/0.394, *p* < 0.001/< 0.001). A correlation comparison showed that the correlation of NLR and PLR with CRP differs during different periods (*p* = 0.037/0.004). Subgroup analysis revealed that the time effect on correlation is related to treatment with Janus kinase inhibitor and anti-interleukin-6 but not antitumor necrosis factors.

## 1. Introduction

Rheumatoid arthritis (RA) is a chronic immune-mediated systemic inflammatory disease characterized by chronic synovitis with pannus formation, leading to joint destruction and disability. The severity of RA varies, and treatment typically involves the use of one or a combination of immunomodulators, commonly referred to as disease-modifying anti-rheumatic drugs (DMARDs) [1]. Acute-phase reactant data, particularly regarding the erythrocyte sedimentation rate (ESR), C-reactive protein (CRP), and the disease activity score 28 (DAS28), are crucial in predicting prognosis and guiding treatment [2,3,4,5]. Disease activity indices are also pivotal in assessing the effectiveness of treatments in clinical trials.

Despite the wide use of ESR and CRP for evaluating disease activity and guiding RA treatment, these markers have limitations [6]. A previous study assessing the correlation of histological evidence with ESR and CRP found that the sensitivities of CRP and ESR in detecting synovial inflammation are only 71.3% and 64.1%, respectively [7]. Meanwhile, DAS28 has been applied in many RA clinical trials to monitor disease activity in RA patients. Inherent confounders are associated with each parameter because the readout scores of tender and swollen joint counts depend on individual patients’ pain tolerances and the examiners’ skills. Additionally, patient global health reports are also subjective [7]. Identifying alternative markers for inflammation would improve the treatment of RA patients.

We sought to find conventional and accessible laboratory tests to increase the detection of inflammatory status in RA patients. Several indices derived from routine complete blood counts with differentials have been evaluated in autoimmune diseases. Hematological indices, such as the neutrophil–lymphocyte ratio (NLR), platelet–lymphocyte ratio (PLR), monocyte–lymphocyte ratio (MLR), platelet–monocyte ratio (PMR), monocyte–neutrophil ratio (MNR), platelet–neutrophil ratio (PNR), and systemic immune-inflammation index (SII), have been recognized as sensitive markers for hidden inflammation and disease activity. A meta-analysis revealed higher NLRs in patients with RA, ankylosing spondylitis (AS), and Behçet’s disease, though not in systemic lupus erythematosus (SLE), when compared to healthy controls [8]. The PLR was also elevated in patients with RA and SLE but not in AS. In another meta-analysis focusing on RA patients, which included 13 NLR studies and 8 PLR studies, NLRs and PLRs were significantly higher in patients with RA [9]. These biomarkers are valuable for assessing RA disease activity. In one study involving RA patients, higher levels of the NLR, PLR, and MLR were observed in the active disease group compared to the remission group, and they could predict treatment response in the active disease group [10]. However, in another study, NLRs and PLRs were significantly higher in the remission group than in the active group [11]. The NLR and PLR are also reported to have a weak correlation with the ESR, CRP, and the DAS28 for distinguishing active and inactive RA. In a multicenter study, hematological indices were significantly higher in patients with RA-associated interstitial lung disease, further underscoring their importance [12].

RA treatments include DMARDs, which are categorized as conventional synthetic DMARDs (csDMARDs), targeted synthetic DMARDs (tsDMARDs), and biological DMARDs (bDMARDs) [13]. TsDMARDs are chemically synthesized drugs targeting specific molecules and have been approved for the treatment of RA. Currently, several Janus kinase inhibitors (JAKis) are available for RA treatment. BDMARDs are categorized based on their targets, including tumor necrosis factor alpha inhibitors (anti-TNF), CD80/CD86 costimulation inhibitors, interleukin-6 inhibitors (anti-IL6), CD20-depleting agents (anti-CD20), and interleukin-1 inhibitors [14]. Patients typically begin treatment with csDMARDs, and in cases of inadequate response, tsDMARDs or bDMARDs are added or substituted [1]. 

In the realm of novel treatments for rheumatoid arthritis, specifically those involving anti-CD20 and JAK inhibitors (JAKis), it is important to note their substantial impacts on lymphocyte and neutrophil counts, as evidenced by previous research [15,16]. However, the influence of these treatments on hematological indices remains an area of limited investigation [17,18,19]. Pereckova showed that RA patients treated with csDMAR have higher NLRs and PLRs compared to healthy donors and patients receiving anti-TNF and anti-IL6. However, the NLRs and PLRs of patients receiving anti-TNF and anti-IL6 are comparable [17]. The effect of treatment duration on these indices was not discussed. Zhou showed that changes in the NLR and PLR from baseline to 6 months after anti-IL6 treatment correlated with changes in the clinical disease activity index and DAS28-ESR. However, they did not examine the difference at three months and did not compare it to other bDMARDs and tsDMARDs [18]. Choe showed that changes in the SII, NLR, and PLR from baseline to 6 months after JAKi treatment correlated with changes in the DAS28-ESR but not with tender or swollen joint counts. Furthermore, the difference in the PLR before and after treatment is insignificant [19]. They also did not examine the correlation at three months. Since RA patients are typically evaluated every three months, such data should be reviewed to see if the correlation of hematologic indices with disease activity is consistent at different time points. Therefore, the aim of this study is to evaluate the correlation between hematological indices and acute-phase reactants before and after treatment with bDMARDs and tsDMARDs at different time points. 

## 2. Materials and Methods

### 2.1. Study Cohort

This single-center retrospective study involved 273 patients with RA who received treatment and follow-up between January 2017 and June 2021 at Hualien Tzu Chi Hospital, a tertiary medical center. All patients met either the 1987 American Rheumatism Association revised criteria for the classification of RA or the American College of Rheumatology/European League Against Rheumatism 2010 RA classification criteria [20,21]. The cohort included RA patients who received their first treatment with tsDMARDs or bDMARDs and were followed up for at least one year. Patients with malignant hematologic diseases or chronic infections were excluded from this study.

This study received approval from the “Research Ethics Committee of Hualien Tzu Chi Hospital, Buddhist Tzu Chi Medical Foundation,” Hualien, Taiwan, with the reference number IRB111-003-B. Written informed consent was waived because the study involved retrospective data analysis. All methods adhered to the relevant guidelines and regulations.

### 2.2. Demographic and Laboratory Data

Demographic information, age, sex, and laboratory data were retrieved from electronic records. Laboratory data included ESR, CRP, and CBC with differentials at three time points: baseline and three and six months after treatment with tsDMARDs or bDMARDs. Hematological indices, specifically NLR, PLR, MLR, and SII, were calculated using data from these time points. SII was calculated by (neutrophil counts × platelet counts)/lymphocyte counts [22].

### 2.3. Statistical Analysis

Data were analyzed using R software version 4.2.1 (R Foundation for Statistical Computing, Vienna, Austria) [23]. Continuous variables were expressed as means ± standard deviations, and categorical variables were represented as frequencies along with their respective percentages. Alterations in laboratory data pre- and post-treatment were assessed using repeated measures analysis of variance (RM-ANOVA). Mauchly’s test for sphericity was performed and Greenhouse–Geisser corrections were used if the result was significant. Eta-squared (η^2^) was also analyzed and described the ratio of variance explained in the dependent variable by a predictor while controlling for other predictors. Cohen’s d was employed to evaluate the treatment effect, providing insight into the effect size at the 3-month interval relative to the pre-treatment period [24].

The relationship between baseline data and ESR response at the 3-month post-treatment mark was examined through ANOVA or Welch ANOVA in the presence of a significant Levene’s test. 

A “good response” was defined as a decrease in ESR by >40% compared to baseline, a “bad response” as a non-decrease or increase in ESR, and intermediate values indicated a mild response. Baseline data were normalized with the Box–Cox transformation using the bestNormalize package version 1.8.3. The lambda values used were 1.132, 1.0506, 1.0126, 1.2142, 1.2054, 1.2612, 1.139, 0.9993, 1.2459, 1.0602, 1.0738, and 1.074 for the ESR, CRP, white blood cell count (WBC), hemoglobin (Hb) level, platelet (PLT) count, monocyte count, neutrophil count, lymphocyte count, NLR, PLR, MLR, and SII, respectively. Dunnett’s test was used for post hoc analysis, with the bad response group as the reference. 

NLR, PLR, MLR, and SII correlations with ESR and CRP were calculated using the Pearson correlation. Correlations were compared using the cocor package version 1.1-4 [25].

## 3. Results

### 3.1. Characteristics and Laboratory Data of Patients before and after Treatment

**Figure 1:**
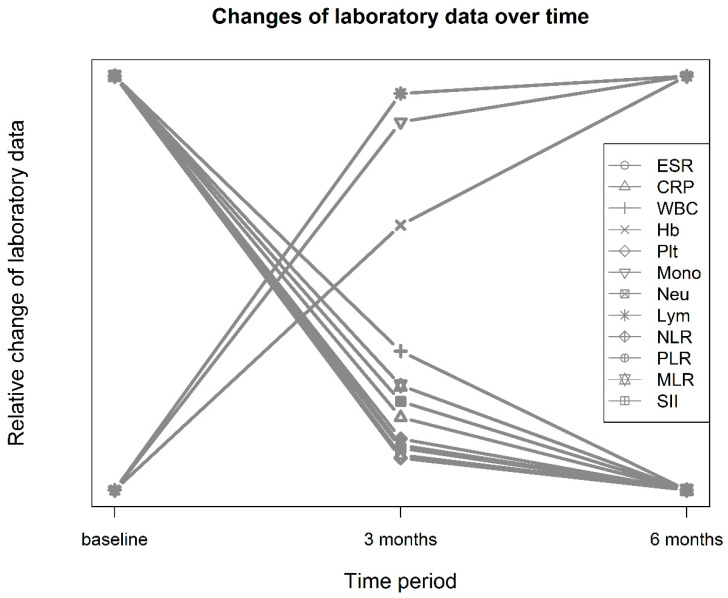
Relative changes in laboratory data before and 3 and 6 months after treatment with target synthetic or biological DMARDs. CRP, C-reactive protein; ESR, erythrocyte sedimentation rate; Hb, hemoglobin; Lym, lymphocytes; Mono, monocytes; MLR, monocyte–lymphocyte ratio; Neu, neutrophils; NLR, neutrophil–lymphocyte ratio; PLR, platelet–lymphocyte ratio; PLT, platelets; SII, systemic immune-inflammation index; WBC, white blood cells.

The study included 273 patients with an average age of 57.2 years, with 74% being female. RM-ANOVA showed significant differences in nearly all laboratory data, except for monocyte counts, obtained before, three months after, and six months after treatment with tsDMARDs or bDMARDs. Mauchly’s test was significant in terms of ESR, CRP, Hb, PLTs, neutrophils, lymphocytes, NLR, MLR, and SII. All results remained significant after Greenhouse–Geisser corrections. Over time, Hb levels, lymphocyte counts, and monocyte counts increased, while all other parameters decreased. Acute-phase reactants, measured by ESR and CRP levels, decreased from baseline to three months and six months after treatment (ESR: 45.9 ± 27.2, 30.0 ± 24.1, and 28.5 ± 22.6 mm/h, *p*-value < 0.001; CRP: 17.0 ± 22.7, 7.74 ± 13.9, and 5.73 ± 10.8 mg/L, *p*-value < 0.001). Hematological indices, including NLR, PLR, MLR, and SII, also significantly decreased across the three time points (NLR: 2.99 ± 1.83, 2.33 ± 1.55, and 2.21 ± 1.47, *p*-value < 0.001; PLR: 187 ± 87.3, 150 ± 76.0, and 146 ± 75.6, *p*-value < 0.001; MLR: 0.300 ± 0.144, 0.273 ± 0.189, and 0.263 ± 0.140, *p*-value = 0.005; SII: 847 ± 615, 600 ± 482, and 571 ± 493, *p*-value < 0.001) (presented in Table 1). Cohen’s d was used then to compare the baseline and three months post-treatment, and η^2^ values are shown in Table 1. Most of these changes occurred around three months after treatment, as shown in Figure 1.

### 3.2. Association of Baseline Data with ESR Response 3 Months after Treatment

**Figure 2:**
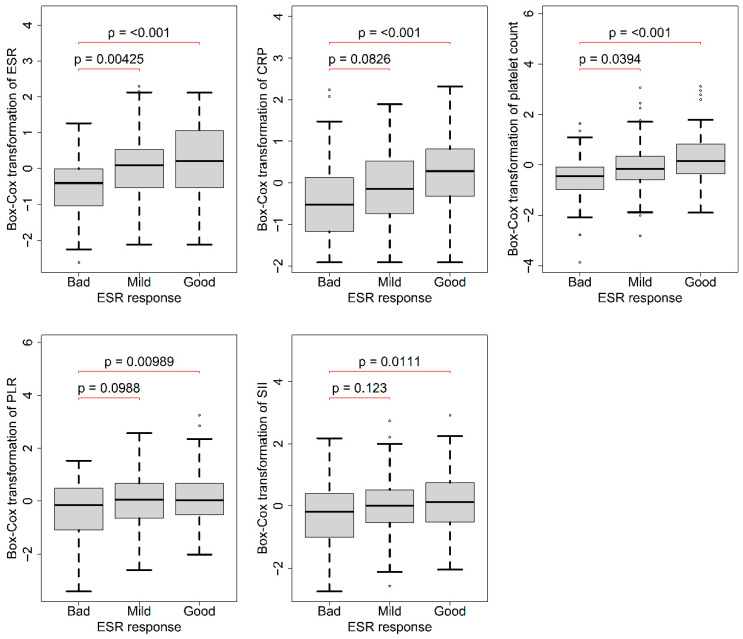
A total of 263 patients whose 3-month ESR available data were analyzed. The ANOVA test showed differences in the baseline ESR, CRP, PLT, PLR, and SII among the three response groups. The post hoc analysis revealed that compared with the bad response group, patients in the good response group had higher baseline ESR, CRP, PLT, PLR, and SII.

A total of 263 patients with available data for 3-month ESR analysis were included, and the results are presented in Table 2. The results of the eta-squared calculations are also shown in Table 2. The ANOVA test showed differences in baseline ESRs, CRP levels, PLT counts, PLRs, and SIIs among the three response groups (all groups showed *p*-values < 0.001). Post hoc analysis revealed that, compared to the bad response group, patients in the good response group had higher baseline ESRs, CRP levels, PLT counts, PLRs, and SIIs (Figure 2).

### 3.3. Correlation of Acute-Phase Reactants with Hematological Indices

Table 3 demonstrates that, at each time point, NLR, MLR, and SII all positively correlated with ESR and CRP, but PLR did not. The correlation of CRP and PLR at 6 months did not reach statistical significance. Comparing correlations revealed that NLR and CRP’s correlation at three months after novel treatment differed from that at the baseline (*p*-value = 0.037). The correlation between CRP, NLR, and SII 3 months post-treatment showed a value of more than 0.3, and the results suggested moderate correlation by the methods of Quinnipiac University [26]. The correlation of PLR with CRP at 6 months also differed from that at the baseline (*p*-value = 0.004), as shown in Table 4.

**Figure 3:**
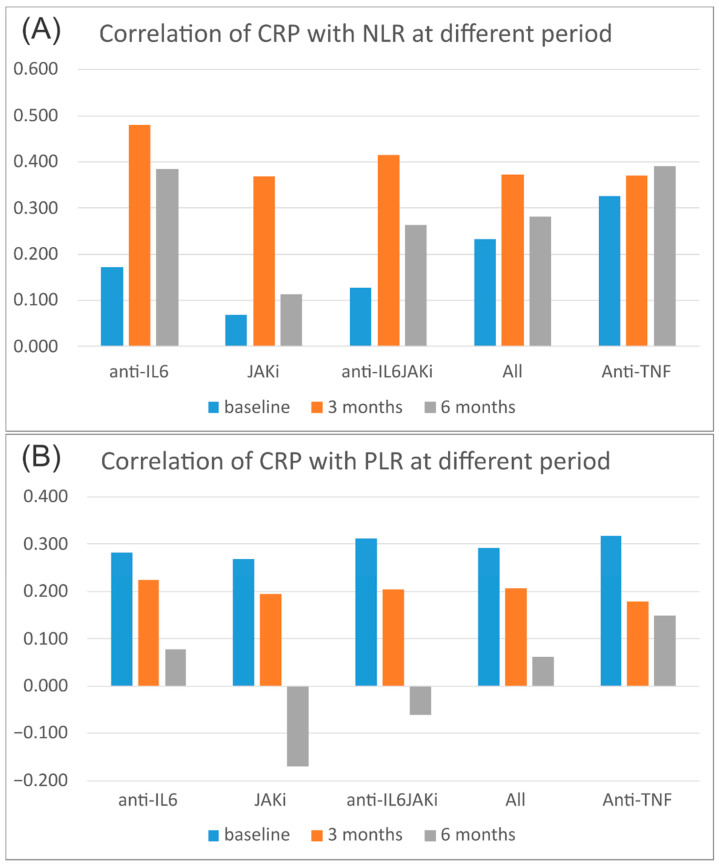
The correlation of PLR with CRP at 6 months also differed from baseline. A subgroup analysis according to the treatment type showed that the correlation of CRP with NLR was stable in patients treated with antitumor necrosis factors but varied a lot in patients treated with JAKis or anti-interleukin-6 receptor antibody (**A**). The correlation of CRP with PLR decreased in all treatment groups and even turned negative in patients treated with JAKis (**B**).

A subgroup analysis based on the treatment type indicated that the correlation of CRP with NLR remained stable in patients treated with anti-TNF but varied considerably in patients treated with JAKis or anti-IL6 (Figure 3A). The correlation of CRP with PLR decreased in all treatment groups and even turned negative in patients treated with JAKis (Figure 3B).

**Figure 4:**
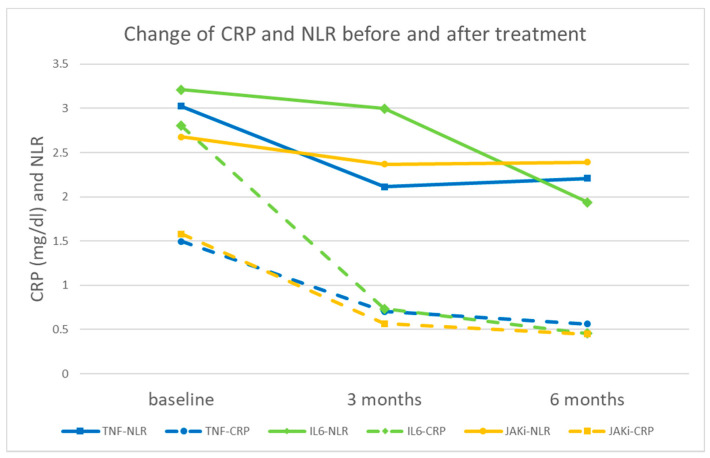
CRP and NLR (neutrophil-to-lymphocyte ratio) of the patients treated with anti-TNF, anti-IL6, and JAK inhibitors at different time points.

In patients treated with anti-IL6 and JAK inhibitors at different time points, the magnitude of changes in NLR and CRP level varied. Patients treated with anti-IL6 showed a less pronounced decrease in NLR in the early stage but a greater decrease in the later stage. Conversely, CRP exhibited a more significant early decrease but a lesser decrease in the later stage, attributed to the inhibitory effect of anti-IL6 on the liver’s CRP production. In patients using JAK inhibitors, the decrease in CRP is comparable to that of anti-TNF, while the reduction in NLR is less apparent. It is speculated that JAK inhibitors themselves may suppress hematopoiesis, interfering with the changes in NLR caused by inflammation (Figure 4).

## 4. Discussion

In our study involving 273 patients, hematological indices, including NLR, PLR, MLR, and SII, exhibited significant differences across the three time points: baseline and 3 and 6 months after treatment. Notably, patients in the good response group had higher baseline ESRs, CRP levels, PLT counts, PLRs, and SIIs compared to the bad response group. A subgroup analysis based on treatment type revealed that the correlation of CRP with NLR remained stable in patients treated with antitumor necrosis factors but showed significant variation in patients treated with JAKis or anti-IL6. Furthermore, the correlation of CRP with PLR decreased in all treatment groups, turning negative in patients treated with JAKis, particularly tofacitinib and tocilizumab.

Hematological indices have been assessed in various autoimmune diseases, with a meta-analysis in autoimmune rheumatic diseases revealing variations in their performance across diseases, such as RA, SLE, AS, Sjögren’s syndrome, Behçet’s disease, and systemic sclerosis [8]. In RA, these markers have been explored for diagnosing RA, monitoring disease activity, and even predicting complications such as interstitial lung disease and stroke [27,28,29,30,31,32]. One previous study included 284 SLE patients and matched them with healthy controls using multivariable linear regression analysis. Hematological indices, such as NLR, MLR, and PRL, were found to be higher in SLE patients. However, the relationship between the various inflammatory scores and disease characteristics was limited [33]. In another cross-sectional study involving 430 patients, the relationship between RA and cardiovascular disease was investigated. Higher values in all hematological composite scores were described as potential inflammatory biomarkers associated with an elevated cardiovascular risk [34].

To the best of our knowledge, our study is the first to focus on the trend of the correlation between hematological indices and ESR and CRP at baseline and 3 and 6 months after treatment. Understanding this trend is crucial to consider the implications of hematological indices for monitoring disease activity and treatment response.

Our study revealed that the NLR, PLR, MLR, and SII gradually decreased following treatment with tsDMARDs or bDMARDs, aligning with the changes observed in the ESR and CRP level. This suggests that these parameters can serve as markers of inflammation. However, it is important to note that the Cohen’s d (standardized mean difference) value of the MLR was much lower compared to other indices. Additionally, there was no significant change in the mean monocyte count before and after treatment. Thus, most of the change in the MLR stemmed from the alteration in the lymphocyte count, indicating that the MLR may not be suitable for monitoring the effects of tsDMARDs or bDMARDs in RA patients. Previous studies on the correlation of MLR with disease activity have also yielded conflicting results [10,11,29].

Our study also found that higher baseline ESR and CRP levels were associated with a greater reduction in ESR levels following treatment. However, among the new indices evaluated, only higher PLR and SII levels were associated with a better ESR response. A higher baseline PLT count was also linked to a better response. This association of PLR and SII with ESR response was primarily attributed to PLT levels. This finding is consistent with a study demonstrating a better efficacy of tocilizumab in RA patients with high PLT levels [35]. Thrombocytosis is known to be associated with chronic RA activity [36], and recent research suggests that platelets not only serve as markers of inflammation but also play a role in mediating inflammation [37].

Biologic treatments can alter cell counts, and this alteration may or may not be linked to treatment response. The effectiveness of rituximab, an anti-CD20 monoclonal antibody, is associated with the depletion of B cells and T cells, with lymphocyte counts gradually recovering within 6 months. A study in RA patients treated with rituximab found that NLR and PLR moderately correlated with DAS28, ESR, and CRP six months after treatment [38].

Interleukin-6 dysregulation is noted in many autoimmune diseases, and treatments targeting the interleukin-6 pathway, such as tocilizumab, have been approved for RA and other autoimmune diseases. In a study on hematological indices, tocilizumab treatment resulted in decreased PLT count, NLR, and PLR six months after treatment [18]. Changes in the PLR correlated with changes in DAS28, but changes in the NLR did not. However, this study did not evaluate the correlation of hematological indices with ESR and CRP, and no longitudinal trend was reported. Janus kinase is essential in the signal transduction of several cytokines, including interleukin-6 and interleukin-23. Several JAKis have been approved for RA treatment. A study evaluating the change in ESR and CRP level with the change in hematological indices six months after JAKi treatment found that changes in SII, NLR, and PLR correlated with changes in ESR, but only changes in SII and PLR correlated with changes in CRP level. In this study, SII, NLR, and PLR failed to predict the response to JAK inhibitors. Furthermore, the correlations of hematological indices with ESR and CRP were evaluated at only one time point (24 weeks after treatment), leaving unanswered questions about their stability at different time points.

Our study found that the correlations of SII and MLR with ESR or CRP remained relatively stable after novel treatments. However, significant differences were observed in the correlation of CRP with NLR and PLR after treatments. Subgroup analysis revealed that these differences were primarily driven by changes in patients treated with anti-IL6 and JAKis. In contrast, in patients treated with anti-TNF, the correlations of CRP with NLR and PLR remained relatively stable. Moreover, these correlations varied at the three time points, suggesting the need for further prospective studies to evaluate the clinical implications of these changes.

One limitation of our study is its retrospective analysis of an observational database, with all enrolled patients originating from a single institution. Additionally, our data lacked complete records of DAS28 due to the retrospective nature of the study. Although DAS28 is crucial for evaluating disease activity and guiding RA treatment, it has its limitations. Our study aimed to explore the correlation of hematological markers with clinical conditions in RA. We observed that RA patients treated with IL6 inhibitors and JAKis exhibited different clinical presentations compared to those treated with anti-TNF drugs. The mechanisms of these drugs may warrant further evaluation concerning their correlation with hematological markers. Given the retrospective nature of our study, which was not a randomized control trial, patients receiving IL6 treatment initially had high CRP levels. Therefore, selection bias remains a limitation.

## 5. Conclusions

Nevertheless, it is crucial to emphasize that the presence of missing data remains a prevalent challenge, and these conventional markers have their limitations when it comes to establishing a strong correlation with the disease activity of RA. In the context of our study, we observed a noteworthy correlation between hematological indices and the acute-phase reactants measured with ESR and CRP level. In our study, hematological indices correlated with ESR and CRP and exhibited substantial decreases after treatment with tsDMARDs and bDMARDs. However, the correlation of CRP with hematological index was influenced by medicine, particularly by JAKis and anti-IL6. This influence varied at different time points. Clinicians should consider this when using hematological indices to monitor treatment response.

## Figures and Tables

**Table 1:** Characteristics and laboratory data of patients before and after treatment.

	Baseline ^a^ (N = 273)	3 Months after ^b^	6 Months after ^c^	F Value	*p*-Value ¶	Cohen’s d *	*η* ^2^
Age (years)	57.2 ± 12.4						
Male: female	71: 202 (26:74%)						
Lab data							
ESR (mm/h)	45.9 ± 27.7	30.3 ± 24.1	28.5 ± 22.6	93.4	<0.001	0.60	0.087
CRP (mg/L)	17.0 ± 22.7	7.74 ± 13.9	5.73 ± 10.8	50.1	<0.001	0.49	0.08
White blood cells (10^9^/L)	6.73 ± 2.28	6.46 ± 2.34	6.35 ± 2.17	4.0	0.019	0.12	0.004
Hemoglobin (g/L)	118 ± 16.4	122 ± 15.8	124 ± 15.6	59.8	<0.001	−0.25	0.028
Platelets (10^9^/L)	277.1 ± 87.1	249 ± 74.5	248 ± 73.8	37.5	<0.001	0.35	0.028
Monocytes (10^9^/L)	0.46 ± 0.20	0.462 ± 0.206	0.463 ± 0.199	0.4	0.691	−0.01	0.000485
Neutrophils (10^9^/L)	4.40 ± 1.99	3.90 ± 1.91	3.76 ± 1.87	14.4	<0.001	0.26	0.019
Lymphocytes (10^9^/L)	1.68 ± 0.63	1.90 ± 0.739	1.93 ± 0.688	26.5	<0.001	−0.32	0.027
Hematological indices							
NLR	2.99 ± 1.83	2.33 ± 1.55	2.21 ± 1.47	26.8	<0.001	0.39	0.042
PLR	187 ± 87.3	150 ± 76.0	146 ± 75.6	46.0	<0.001	0.45	0.049
MLR	0.300 ± 0.144	0.273 ± 0.189	0.263 ± 0.140	5.5	0.006	0.16	0.008
SII	847 ± 615	600 ± 482	571 ± 493	33.9	<0.001	0.45	0.05

Abbreviations: CRP, C-reactive protein; ESR, erythrocyte sedimentation rate; MLR, monocyte–lymphocyte ratio; NLR, neutrophil–lymphocyte ratio; PLR, platelet–lymphocyte ratio; SII, systemic immune-inflammation index. ^a^—missing data: CRP 1, ^b^—missing data: ESR 10, CRP 13, all other 8, ^c^—missing data: ESR 13, CRP 13, white blood cell/hemoglobin/platelet 11, all other 12, ¶—*p*-values after Greenhouse–Geisser corrections: ESR, CRP, Hb, PLT, neutrophil, lymphocyte, NLR, MLR, and SII. * Cohen’s d between baseline and 3 months after.

**Table 2:** Association of baseline data with ESR response 3 months after treatment.

	Good (N = 129)	Mild (N = 81)	Bad (N = 53)	*p*-Value *	*η*^2^ *
Lab data					
ESR (mm/h)	50.9 ± 29.4	46.4 ± 26.7	33.0 ± 21.8	<0.001 **	0.0600
CRP (mg/L)	20.8 ± 24.5	14.8 ± 19.3	12.5 ± 23.6	<0.001 **	0.0700
WBC (10^9^/L)	6.91 ± 2.28	6.66 ± 2.51	6.43 ± 1.96	0.404	0.0070
Hb (g/L)	119 ± 15.2	115 ± 15.3	120 ± 19.2	0.135	0.0200
PLT (10^9^/L)	298 ± 85.7	266 ± 86.3	234 ± 76.1	<0.001 **	0.0900
Monocyte (%)	0.467 ± 0.20	0.443 ± 0.20	0.445 ± 0.18	0.524	0.0050
Neutrophil (%)	4.54 ± 1.95	4.44 ± 2.26	4.13 ± 1.76	0.405	0.0069
Lymphocyte (%)	1.71 ± 0.61	1.60 ± 0.57	1.69 ± 0.77	0.445	0.0062
Hematological indices					
NLR	2.91 ± 1.48	3.17 ± 2.16	3.05 ± 2.16	0.706	0.0058
PLR	196 ± 91.0	187 ± 87.8	164 ± 75.2	0.021 **	0.0300
MLR	0.30 ± 0.15	0.297 ± 0.14	0.297 ± 0.14	0.993	0.0001
SII	895 ± 603	866 ± 676	725 ± 571	0.023 **	0.0300

CRP, C-reactive protein; ESR, erythrocyte sedimentation rate; WBC, white blood count; Hb, hemoglobulin; PLT, platelet count; MLR, monocyte–lymphocyte ratio; NLR, neutrophil–lymphocyte ratio; PLR, platelet–lymphocyte ratio; SII, systemic immune-inflammation index. * Data presented as original data but *p*-value and eta-squared calculated based on Box–Cox transformed data. One missing datum (CRP) in the good response group, ** *p*-value < 0.05.

**Table 3:** Correlation of NLR, PLR, MLR, and SII with ESR and CRP at each time point.

		Correlation				*p*-Value			
		NLR	PLR	MLR	SII	NLR	PLR	MLR	SII
ESR	Baseline	0.141	0.273	0.206	0.226	0.020	<0.001	<0.001	<0.001
	3 months	0.211	0.250	0.177	0.227	<0.001	<0.001	0.004	<0.001
	6 months	0.160	0.170	0.134	0.179	0.0096	0.006	0.031	0.004
CRP	Baseline	0.233	0.291	0.187	0.361	<0.001	<0.001	0.002	<0.001
	3 months	0.373	0.207	0.250	0.394	<0.001	<0.001	<0.001	<0.001
	6 months	0.280	0.062	0.187	0.313	<0.001	0.318	0.003	<0.001

CRP, C-reactive protein; ESR, erythrocyte sedimentation rate; MLR, monocyte–lymphocyte ratio; NLR, neutrophil–lymphocyte ratio; PLR, platelet–lymphocyte ratio; SII, systemic immune-inflammation index.

**Table 4:** Comparison of the correlation of NLR, PLR, MLR, and SII with ESR and CRP at each time point.

	Comparison of Correlation at Baseline with	NLR *p*-Value	PLR *p*-Value	MLR *p*-Value	SII *p*-Value
ESR	3 months	0.238	0.826	0.782	0.827
	6 months	0.706	0.073	0.328	0.509
CRP	3 months	0.037	0.310	0.329	0.537
	6 months	0.418	0.004	0.964	0.464

CRP, C-reactive protein; ESR, erythrocyte sedimentation rate; MLR, monocyte–lymphocyte ratio; NLR, neutrophil–lymphocyte ratio; PLR, platelet–lymphocyte ratio; SII, systemic immune-inflammation index.

## Data Availability

The datasets generated and analyzed during the current study are not publicly available but are available from the corresponding author upon reasonable request.
